# Dysphagia Revealing Pulmonary Squamous Cell Carcinoma: Successful Palliation With Esophageal Stenting in an Elderly Patient

**DOI:** 10.1002/ccr3.72370

**Published:** 2026-04-24

**Authors:** Mehdi Belaich, Amine Bentayeb, Mohamed El Mazghi, Aymane Assili, Mehdi Zouaoui, M'barek Azouaoui, Youssef Hnach, Nourdin Aqodad

**Affiliations:** ^1^ Department of Gastroenterology Centre Hospitalo‐Universitaire Mohammed VI Agadir Morocco; ^2^ Department of Gastroenterology Hopital Militaire Oued Eddahab Inzegane Morocco

**Keywords:** dysphagia, endoscopy, esophageal stenosis, lung neoplasms, palliative care, stents

## Abstract

In elderly patients, a smooth concentric esophageal stricture with normal mucosa should raise suspicion of extraesophageal causes, prompting cross‐sectional imaging such as computed tomography. Dysphagia may rarely be the initial symptom of lung cancer, and esophageal stenting offers rapid, effective palliation, restoring oral intake, and improving quality of life.

## Graphical abstract

Fluoroscopic control after deployement of a partially covered esophageal self‐expandable metallic stent (12.5 cm × 20 mm), demonstrating adequate expansion across the stenotic segment.

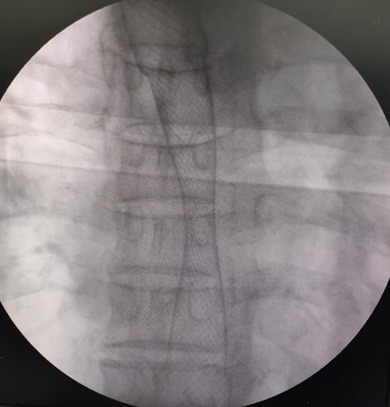

## 1 Introduction

Dysphagia is a frequent complaint in gastroenterology, most often caused by intrinsic esophageal pathology such as carcinoma, strictures, or motility disorders. Its occurrence as the initial presentation of lung cancer is rare, estimated at only 1%–2% of cases [1]. Mechanisms include extrinsic compression by tumors or lymphadenopathy, direct esophageal invasion, and treatment‐related stenosis [2].

Esophageal self‐expandable metallic stents (SEMS) are widely employed to palliate malignant dysphagia. While most data concern esophageal carcinoma, SEMS also provide effective palliation in dysphagia caused by mediastinal or pulmonary malignancies [3].

We present the case of an elderly patient in whom dysphagia was the revealing symptom of a primary pulmonary squamous cell carcinoma with mediastinal involvement. The case illustrates the importance of cross‐sectional imaging in the evaluation of unexplained strictures and demonstrates the efficacy of SEMS placement for palliation.

## 2 Case History/Examination

An 82‐year‐old man, with a history of 30 pack‐year smoking (quit 10 years earlier), ischemic heart disease managed medically with aspirin, beta‐blocker, and statin, and end‐stage renal disease on hemodialysis for five years, presented with a six‐month history of progressively worsening dysphagia. The symptom initially affected solids, gradually worsened, and extended to liquids during the month prior to admission, ultimately resulting in complete aphagia and an associated weight loss of 15 kg. He also reported a two‐month history of chronic productive cough. Physical examination showed a stable patient with no palpable cervical lymphadenopathy. Cardiopulmonary and abdominal examinations were unremarkable.

## 3 Differential Diagnosis, Investigations and Treatment

Given the progressive dysphagia to both solids and liquids associated with significant weight loss, a malignant etiology of esophageal obstruction was strongly suspected. The differential diagnosis included primary esophageal carcinoma, extrinsic esophageal compression due to mediastinal pathology, and, less likely, benign esophageal stricture.

**FIGURE 1:**
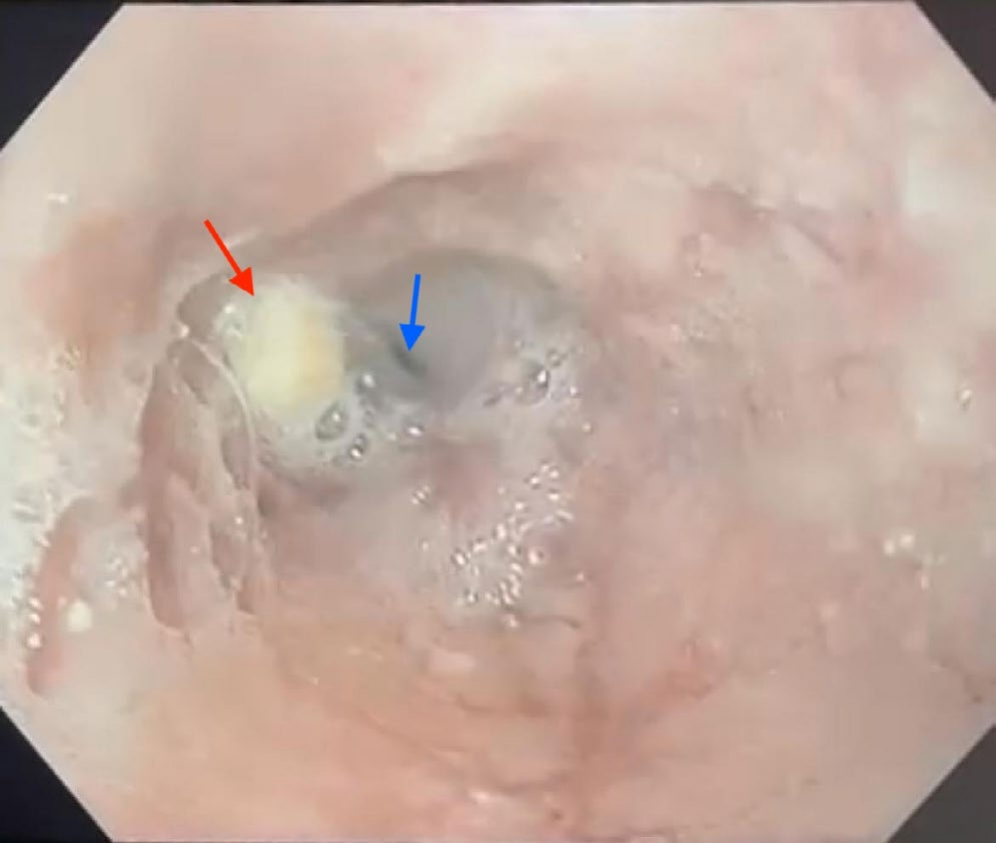
Upper gastrointestinal endoscopy revealing a tight, concentric mid‐esophageal stricture located 25 cm from the dental arches (blue arrow), associated with food impaction (red arrow).

Esophagogastroduodenoscopy revealed a tight, concentric mid‐esophageal stricture beginning 25 cm from the dental arches. The mucosa was smooth, without inflammation or ulceration. Food impaction was noted, and the stenosis could not be traversed with a standard endoscope (Figure 1).

Thoraco‐abdominal computed tomography (CT) demonstrated a right mediastino‐pulmonary lesion measuring 42 × 36 mm, with irregular contours and partial encasement of the azygos vein and the esophagus over 180°. The mass abutted the right main and lower lobar bronchi, the right atrial appendage, and the vertebral body at T8. Additional findings included apical emphysema, mediastinal lymphadenopathy up to 10 mm, and thoracic aortic atheromatosis.

Bronchoscopy revealed infiltrative thickening of the right bronchus intermedius and middle lobe bronchus, with narrowing of the right middle lobe bronchus. Biopsies and bronchial washings were obtained. Histopathological examination confirmed non‐small cell lung carcinoma of squamous type.

**FIGURE 2:**
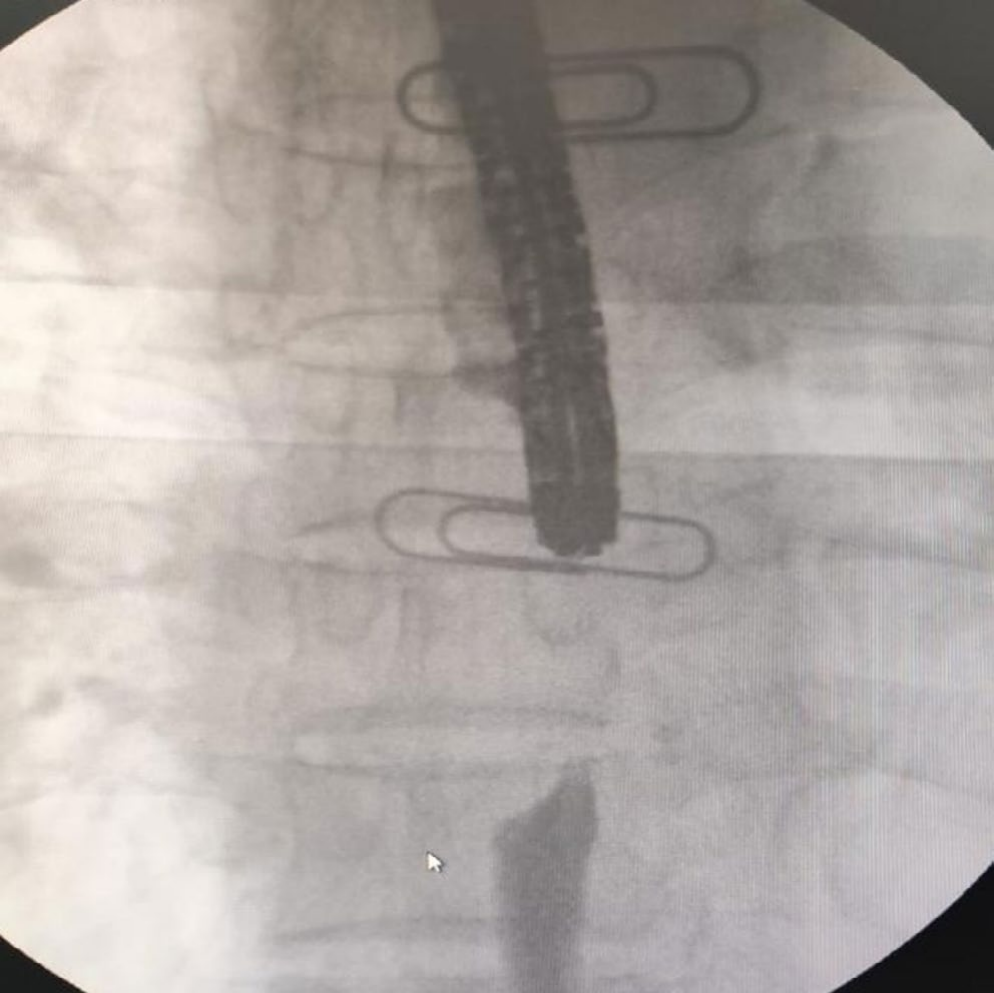
Contrast esophagogram showing a severe concentric mid‐thoracic esophageal stricture prior to stent placement.

**FIGURE 3:**
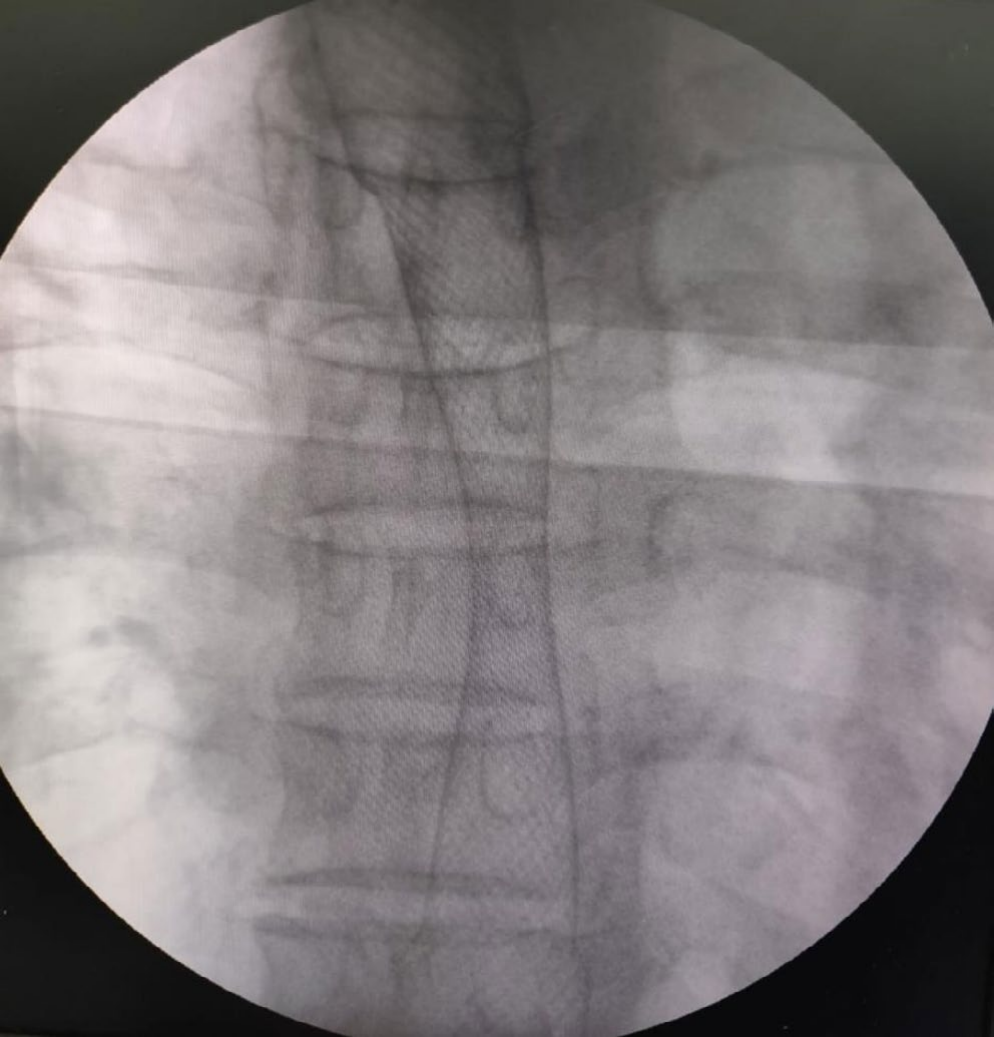
Fluoroscopic control after deployment of a partially covered esophageal self‐expandable metallic stent (12.5 cm × 20 mm), demonstrating adequate expansion across the stenotic segment.

**FIGURE 4:**
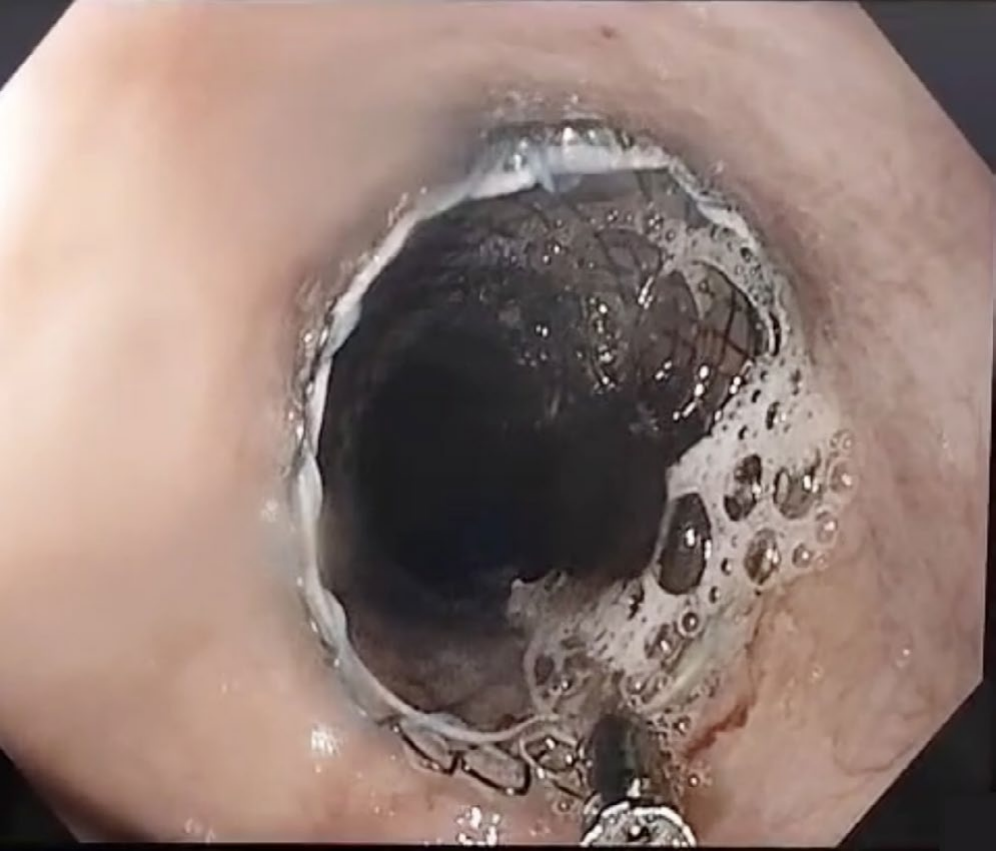
Endoscopic view following deployment of the partially covered esophageal self‐expandable metallic stent demonstrating adequate stent expansion and luminal patency.

Endoscopic extraction of the impacted food bolus was first performed, followed by an intraoperative esophagogram, which revealed a concentric, tight mid‐esophageal stricture extending over 25 mm (Figure 2). Based on these findings, a partially covered SEMS (12.5 cm × 20 mm) was deployed under combined endoscopic and fluoroscopic guidance. A subsequent esophagogram confirmed complete stent expansion across the stricture, with optimal positioning and restoration of luminal patency (Figure 3). Immediate endoscopic assessment further demonstrated satisfactory expansion of the prosthesis with clearance of the esophageal lumen (Figure 4).

## 4 Conclusion and Results (Outcome and Follow‐Up)

The procedure resulted in rapid resolution of dysphagia, with no peri‐procedural complications and resumption of oral intake within 24 h. Follow‐up evaluation included clinical assessment of swallowing function, weight monitoring, nutritional status, and confirmation of appropriate stent positioning on control imaging, all of which were satisfactory. At one‐month follow‐up, the patient remained asymptomatic, tolerated oral feeding, and had regained 5 kg.

## 5 Discussion

Dysphagia is a common symptom encountered in gastroenterology, but cases resulting from extraesophageal disease remain uncommon and may be easily overlooked. Although uncommon, dysphagia is reported in approximately 3%–4% of patients with lung cancer as a result of esophageal involvement through various mechanisms, yet it constitutes the initial presenting manifestation in only 1%–2% of cases [1, 2]. The underlying mechanisms include direct tumor invasion of the esophagus, extrinsic compression by the primary lesion or mediastinal lymphadenopathy, and, less frequently, post‐treatment fibrosis [2]. Among these mechanisms, pure extrinsic compression without mucosal involvement, as in our patient, represents a particular diagnostic challenge, since endoscopy may show only a concentric narrowing with an otherwise normal‐appearing mucosa. In such circumstances, extrinsic etiologies should be systematically considered. Cross‐sectional imaging is crucial to identify mediastinal masses or pulmonary lesions responsible for esophageal compression. In our patient, contrast‐enhanced CT clearly demonstrated a mediastino‐pulmonary mass encasing the esophagus, leading to diagnostic suspicion of a primary pulmonary malignancy with mediastinal involvement.

When bronchial involvement is suspected or when a thoracic lesion is accessible via the airways, bronchoscopy offers direct visualization and tissue sampling, enabling histological confirmation. For mediastinal lymph node assessment, endobronchial ultrasound‐guided needle aspiration (EBUS‐TBNA) is particularly valuable, whereas CT‐guided transthoracic biopsy is more suitable for peripheral parenchymal lesions not reachable endoscopically [4]. In our case, bronchoscopy with biopsy was sufficient to establish the diagnosis of squamous cell carcinoma, highlighting its central role when endobronchial involvement is present, while other modalities remain valuable in different anatomical contexts.

SEMS have become the cornerstone of palliative therapy for malignant dysphagia, providing rapid relief and improving quality of life [5]. SEMS are the preferred therapeutic option in patients with advanced disease and limited life expectancy, as they provide immediate mechanical relief of obstruction. Evidence from randomized controlled trials indicates that SEMS offer faster improvement of swallowing compared with intraluminal brachytherapy (ILBT), owing to their instant therapeutic effect once full expansion is achieved, usually within 24–48 h after deployment. Consequently, SEMS are particularly suitable in patients with a poor prognosis, where rapid restoration of oral intake and quality of life are prioritized. Conversely, in patients with a relatively favorable prognosis, ILBT may offer more durable relief of dysphagia, although at the cost of a delayed onset of benefit [5]. Thus, treatment choice should be guided by prognosis, anticipated survival, and overall treatment goals within a multidisciplinary framework.

Although most evidence comes from primary esophageal cancer, several studies have demonstrated SEMS efficacy in extrinsic esophageal compression due to mediastinal or pulmonary malignancies. In a series of 56 patients with lung‐cancer–related dysphagia, symptomatic improvement was reported in 96.4% of cases following SEMS placement, with mean post‐stenting survival of 4.3 months in non‐fistula cases and 2.8 months in those with tracheoesophageal fistula [6]. Other studies confirm that SEMS provide effective palliation in malignant dysphagia caused by extrinsic compression, with rapid improvement of swallowing and acceptable complication rates [7]. However, when tracheobronchial involvement is significant, manifested by airway compression or tracheoesophageal fistula, a combined approach may be necessary. Reports of double stenting, with both airway and esophageal prostheses, show that the technique is feasible, provides additional symptom relief, and can improve quality of life in carefully selected patients [8, 9].

Although SEMS are highly effective, their use can be associated with both early and late adverse events. In the short term, patients may experience gastroesophageal reflux, retrosternal discomfort, bleeding, aspiration pneumonia, or, less commonly, perforation, with reported frequencies ranging from 3% to 9% [5]. Over time, complications tend to shift, with reflux and persistent chest pain each occurring in roughly 15% of cases, hemorrhage in about 10%, aspiration pneumonia in a similar proportion, and fistula formation in approximately 5%. Recurrent dysphagia may also arise due to tumor ingrowth or stent migration [5]. Several series have noted that complication rates appear to be increasing, possibly reflecting the broader use of chemo‐radiotherapy before stent placement, which may render the esophageal wall more fragile [5].

Overall, the selection of the palliative strategy must be multidisciplinary, integrating gastroenterology, pulmonology, radiology, and oncology expertise. The choice between isolated esophageal stenting or combined stenting should be guided by the anatomical pattern of obstruction, the presence of airway compromise, and the patient's overall condition.

This case highlights that dysphagia, although most often related to primary esophageal disease, may occasionally represent the initial manifestation of lung cancer with mediastinal involvement. When endoscopic evaluation reveals a concentric esophageal stricture without visible mucosal abnormalities, extrinsic causes should still be considered. In such situations, thoracic imaging is warranted, particularly in elderly patients presenting with significant weight loss and risk factors such as a history of smoking. Esophageal self‐expandable metallic stents provide a rapid, safe, and effective palliative option, even in patients with significant comorbidities, allowing resumption of oral intake and meaningful improvement in quality of life. Optimal management therefore requires a multidisciplinary approach to evaluate both esophageal and airway involvement and to anticipate situations where combined stenting may be required.

## Author Contributions

**Mehdi Belaich:** conceptualization, investigation, methodology, visualization, writing – original draft. **Amine Bentayeb:** investigation. **Mohamed El Mazghi:** investigation. **Aymane Assili:** writing – review and editing. **Mehdi Zouaoui:** validation, writing – review and editing. **M'barek Azouaoui:** methodology, validation. **Youssef Hnach:** investigation, supervision, validation, writing – review and editing. **Nourdin Aqodad:** project administration, supervision, validation, writing – review and editing.

## Funding

The authors have nothing to report.

## Ethics Statement

This case report was conducted in accordance with the ethical standards of the institution and with the principles of the Declaration of Helsinki.

## Consent

Written informed consent was obtained from the patient for the publication of this case report and all accompanying images.

## Conflicts of Interest

The authors declare no conflicts of interest.

## Data Availability

Data sharing not applicable to this article as no datasets were generated or analysed during the current study.
